# Research on Multiple-Factor Dynamic Constitutive Model of Poured Asphalt Concrete

**DOI:** 10.3390/ma17153804

**Published:** 2024-08-01

**Authors:** Jianxiang Wang, Xinjun Tang, Qin Wu, Chuanxiang Chen

**Affiliations:** 1 School of Civil Engineering and Architecture, Guizhou Minzu University, Guiyang 550025, China; wuqin0508@163.com (Q.W.); ccx039@163.com (C.C.); 2 College of Hydraulic and Civil Engineering, Xinjiang Agricultural University, Urumqi 830052, China; wlmqwjx124@163.com

**Keywords:** dynamic characteristics, influencing factor, dynamic elasticity modulus, damping ratio

## Abstract

This study conducted dynamic triaxial tests on a typical poured asphalt concrete material of core walls in Xinjiang, exploring the dynamic characteristics of poured asphalt concrete under various confining pressures, principal stress ratios, and vibration frequencies. On this basis, the dynamic constitutive relationship of poured asphalt concrete was investigated using the Hardin–Drnevich model. The results indicate that under different confining pressures, principal stress ratios, and vibration frequencies, the variation patterns of the backbone lines of dynamic stress-strain of poured asphalt concrete are basically identical, consistent with a hyperbolic curve. The confining pressure and principal stress ratio significantly affect the backbone line of dynamic stress-strain. By comparison, frequency has a minimal effect. The changing trends of dynamic elasticity modulus and damping ratio of poured asphalt concrete under various factors are almost the same. When the material has high dynamic stress and strain, the hysteresis loop is large. When the curve of the damping ratio becomes flat, the asymptotic constant can be used as the maximum damping ratio. The relationship between the reciprocal of the dynamic elasticity modulus and the dynamic strain of poured asphalt concrete exhibits a linear distribution. Under different ratios of confining pressure to principal stress, there are large discrepancies between the calculated values from the formula and the experimental fitting values of the maximum dynamic elasticity modulus, and the maximum relative errors reach 16.65% and 18.15%, respectively. Therefore, the expression for the maximum dynamic elasticity modulus was modified, and the calculated values using the modified formula were compared with the experimental fitting values. The relative errors are significantly reduced, and the maximum relative errors are 3.02% and 2.04%, respectively, in good agreement with the fitting values of the experimental data. The findings of this article render a theoretical basis and reference for the promotion and application of poured asphalt concrete.

## 1. Introduction

From the perspective of construction methods, asphalt concrete core walls can be divided into roller-compacted and poured [1,2,3]. The amount of asphalt used in roller-compacted asphalt concrete core walls is small, generally between 6% and 8% [4,5,6]. The mixture is loose, necessitating paving and compaction to achieve the required permeability and strength, which highly changes construction equipment [7,8,9,10]. In contrast, poured asphalt concrete construction dispenses with rolling or vibration of the concrete mixture. At a certain temperature (150–160 °C), the concrete mixture can be directly poured and spread into the warehouse formed by the formwork or trench, level itself with its fluidity, and compact under its volume weight. Poured asphalt concrete is characterized by solid crack resistance, high-pressure impermeability, strong crack self-healing ability, and eminent covariant capacity [11,12]. Pouring construction is fast, easy to manage, and convenient for quality control, thus ensuring pouring quality. Therefore, it has broad application in the anti-seepage construction of rock-fill dams in frigid regions [13,14]. Located in the hinterland of the Eurasian continent, Xinjiang is one of the earthquake-prone areas in China [15]. It is of significant importance to probe the dynamic characteristics and constitutive model of poured asphalt concrete core wall dams in seismic-prone regions such as Xinjiang.

Global research on the dynamic constitutive models of core wall asphalt concrete materials mainly includes equivalent linear models [16,17,18,19] and elastic-plastic dynamic constitutive models [20,21,22,23]. The former has been widely used due to the simplicity of parameters and easy acquisition through experiments. To reveal the dynamic characteristics of asphalt core embankment dams, scholars carried out a dynamic triaxial experiment on asphalt concrete under different temperatures and stress states [24,25]. The results indicate that the maximum dynamic elastic modulus of asphalt concrete increased with decreasing temperature, increasing principal stress ratio, and increasing confining pressure. However, the damping ratio showed the opposite trend [26]. Based on the features of equivalent linear models, some scholars used consolidation ratio, confining pressure, and temperature as model parameters to establish dynamic stress-strain relationships that can reflect the changes in the elastic modulus of asphalt concrete with these parameters [27,28,29,30]. Some discussed the reliability of anti-seepage structures for asphalt mixtures, demonstrating their satisfying deformation capacity and seismic performance [31,32]. Some performed solitary and cyclic triaxial tests on asphalt concrete core wall materials with asphalt content between 5.5% and 7.0%, obtaining shear moduli under different asphalt content, confining stresses, and principal stress ratios [33,34]. Equivalent linear models play a crucial role in the design and safety evaluation of asphalt concrete core walls [35,36,37]. The above analysis indicates that existing research concentrates on the dynamic characteristics and dynamic constitutive models of roller-compacted asphalt concrete, verifying the feasibility and effectiveness of equivalent linear models for roller-compacted asphalt concrete core walls. This provides a reference for exploring the constitutive models of poured asphalt concrete materials.

The studies on poured asphalt concrete can be summarized into the following two aspects: (1) Mix design. It has been reported that when choosing poured asphalt concrete, the physical properties of asphalt and the construction environment are essential considerations. In terms of filler selection, the surface activity and hydrophilicity of the material should be comprehensively assessed. When choosing coarse and fine aggregates, the impact of aggregates on technical performance should be considered. Regarding the determination of the mix proportion of poured asphalt concrete, first, the principle of maximum density should be adopted; second, the method of selecting high-density mixtures based on particle size coefficients can be used [38,39,40]. According to the principle of mix design, the mix design methods of poured asphalt concrete mixture were studied, and the feasibility was verified through specific engineering examples [41,42]. (2) Mechanical performance testing and numerical simulation. Some researchers conducted triaxial tests on poured asphalt concrete specimens with different asphalt content using indoor triaxial apparatus, analyzing the effects of asphalt content and other factors on the modulus coefficient K, internal friction angle φ, cohesive force C, and the maximum deviatoric stress [43,44].

In summary, studies on roller-compacted asphalt concrete center around static and dynamic analyses, and research on poured asphalt concrete focuses on materials, mix proportions, and static performance experimental analysis. However, the attention paid to the dynamic characteristics and constitutive models of poured asphalt concrete is insufficient. Given this, this study conducted dynamic triaxial tests on a typical core wall poured asphalt concrete material to explore the changes in the backbone line of dynamic stress-strain, dynamic elasticity modulus, and damping ratio, as well as the effects of various confining pressures, principal stress ratios, and vibration frequencies on the dynamic characteristics of the material. Finally, a constitutive model of core wall poured asphalt concrete materials was built using the Hardin–Drnevich model.

## 2. Materials and Methods

### 2.1. Experimental Materials and Equipment

The dynamic characteristic test adopted the 70 # asphalt produced by Karamay Petrochemical Company (Karamay, China). The coarse and fine aggregates were natural gravel materials in the dam area, and the filler was cement. The quality mix proportions of poured asphalt concrete materials are shown in Table 1. The asphalt dosage (the ratio of asphalt material mass to total mineral aggregate mass) is 9%. The properties of the raw materials are shown in Table 2, Table 3, Table 4 and Table 5.

**Figure 1:**
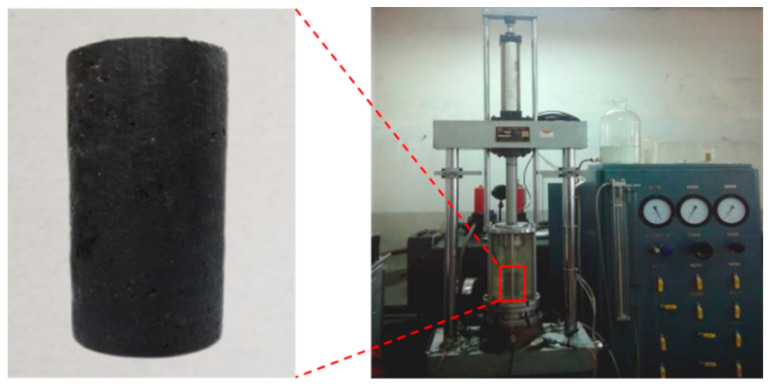
Vibration triaxial instrument and specimen.

According to the quality mix proportions, the specimens were prepared using the isostatic-pressing method. The dimension of poured asphalt concrete specimens is Φ 80 × 160 mm, with a deviation of ±2 mm. The specific parameters are shown in Figure 1. The experiment used a TAJ-20 vibration triaxial apparatus (Figure 1). It can exert vibration loads of multiple forms and intensities to measure the dynamic stress and strain of the specimen under vibration, which can be used to make qualitative and quantitative judgments on the changes in relevant indicators of the material. This instrument adopted two sets of electro-hydraulic servo closed-loop control systems, which had good amplitude-frequency characteristics and rapid frequency response. It can perform static and dynamic triaxial tests on the sample and realize unidirectional or bi-directional excitation.

### 2.2. Experimental Content and Scheme

In this test, the load was applied in the form of a sine wave, and stress was controlled in the axial direction. The dynamic load was divided into ten levels for stepwise application, with twenty vibrations for each level. The variation patterns of dynamic stress, dynamic elasticity modulus, and damping ratio with dynamic strains of core wall poured asphalt concrete were investigated, as well as the effects of confining pressure, principal stress ratio, and vibration frequency on the dynamic stress, dynamic elasticity modulus, and damping ratio of the material. Based on the research results, a dynamic constitutive model of poured asphalt concrete was established (Table 6).

### 2.3. Hardin-Drnevich Model

**Figure 2:**
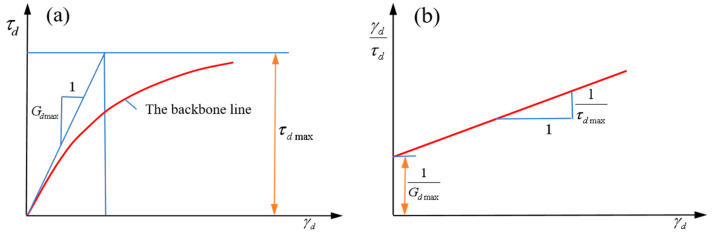
Hardin–Drnevich model. (**a**) The backbone line; (**b**) The relationship between $\gamma_{d}/\tau_{d}$ and $\gamma_{d}$.

The Hardin–Drnevich model assumes that the trunk line is a hyperbola [45], as shown in Figure 2a and Formula (1).

(1) $$\tau_{d}=\frac{\gamma_{d}}{a+b\gamma_{d}}$$

(2) $$\frac{\gamma_{d}}{\tau_{d}}=b\gamma_{d}+a$$

In the formula: $\tau_{d}$: the dynamic shear stress, $P_{a}$; $\gamma_{d}$: the dynamic shear strain, dimensionless; a, b: experimental parameters.

In Figure 2a, when the dynamic shear strain γ→∞, the asymptote of the hyperbolic curve is the ultimate shear stress $\tau_{d\max}$. When γ=0, the tangent slope of the hyperbola is the maximum shear modulus $G_{d\max}$. After coordinate transformation, the relationship line of $\gamma_{d}/\tau_{d}\sim \gamma_{d}$ was plotted, as shown in Figure 2b. It is a straight line, with the intercept $a=1/G_{d\max}$ and slope $b=1/\tau_{d\max}$ on the vertical axis. Therefore, formula (2) can be expressed as:

(3) $$\frac{\gamma_{d}}{\tau_{d}}=\frac{\gamma_{d}}{\tau_{d\max}}+\frac{1}{G_{d\max}}$$

Therefore, the equivalent linear shear modulus $G_{eq}$ can be expressed as:

(4) $$G_{eq}=\frac{\tau_{d}}{\gamma_{d}}=\frac{G_{d\max}}{1+\frac{\gamma_{d}}{\gamma_{r}}}$$

In the formula: $\gamma_{r}$: the reference shear strain, $\gamma_{r}=\tau_{d\max}/G_{d\max}$, dimensionless; $G_{d\max}$: the maximum dynamic shear modulus, $P_{a}$.

The equivalent damping ratio $D_{eq}$ of the Hardin–Drnevich model can be expressed as:

(5) $$D_{eq}=D_{\max}\frac{\gamma_{d}/\gamma_{r}}{1+\gamma_{d}/\gamma_{r}}$$

In the formula: $D_{\max}$: a test parameter, representing the maximum damping ratio, dimensionless.

## 3. Results and Discussion

### 3.1. Test Results and Analysis

#### 3.1.1. Dynamic Stress

**Figure 3:**
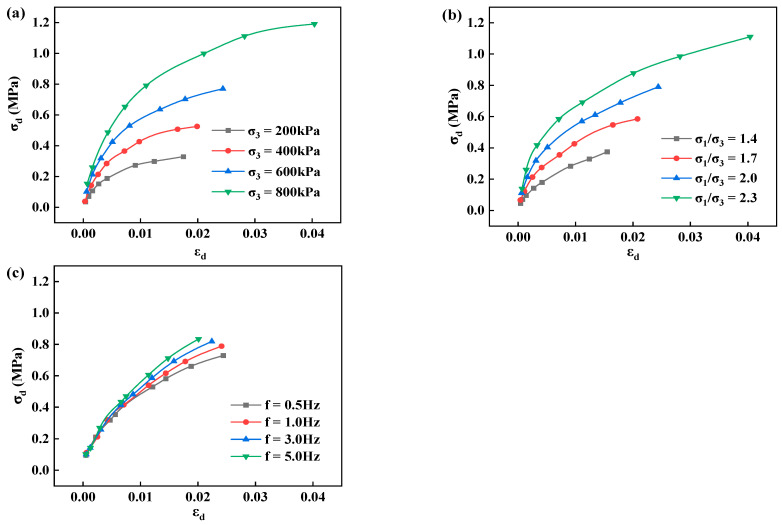
Backbone lines of dynamic stress-strain of poured asphalt concrete under various influencing factors. (**a**) confining pressure; (**b**) principal stress ratio; (**c**) vibration frequency.

According to the testing plan, the dynamic triaxial test was conducted to identify the changing pattern of dynamic stress ($\sigma_{d}$)—strain ($\varepsilon_{d}$) of poured asphalt concrete, as well as the effects of confining pressure ($\sigma_{3}$), principal stress ratio ($\sigma_{1}/\sigma_{3}$), and vibration frequency (f) on the dynamic stress-strain curve, as shown in Figure 3.

Under the impacts of confining pressure, principal stress ratio, and vibration frequency, the backbone lines of dynamic stress-strain exhibit similar upward trends. When the dynamic strain increases from 0.006 to 0.016, the dynamic stress rises from 0.101 MPa to 0.216 MPa. As the load climbs, the dynamic stress and strain gradually grow. When the dynamic strain ascends from 0.0134 to 0.0178, the dynamic stress increases by 0.066 MPa, and the backbone line of dynamic stress-strain tends to flatten, which is consistent with a hyperbolic change. Therefore, a hyperbolic curve can be employed to characterize the variation pattern of the backbone line of dynamic stress-strain of core wall poured asphalt concrete. Under the same dynamic strain, poured asphalt concrete significantly elevates with the confining pressure and principal stress ratio, indicating that they play a prominent role in the backbone line of dynamic stress-strain. The dynamic strength profoundly enlarges with the two indicators, implying that the stiffness of the material rises under high confining pressure and principal stress ratio, gradually leaning toward the stress axis. Vibration frequency barely affects the backbone lines of dynamic stress-strain, which concentrate in a narrow band and fluctuate slightly. Under the same dynamic strain, high vibration frequencies are a little greater than the dynamic stress at low frequencies.

#### 3.1.2. Dynamic Elasticity Modulus

**Figure 4:**
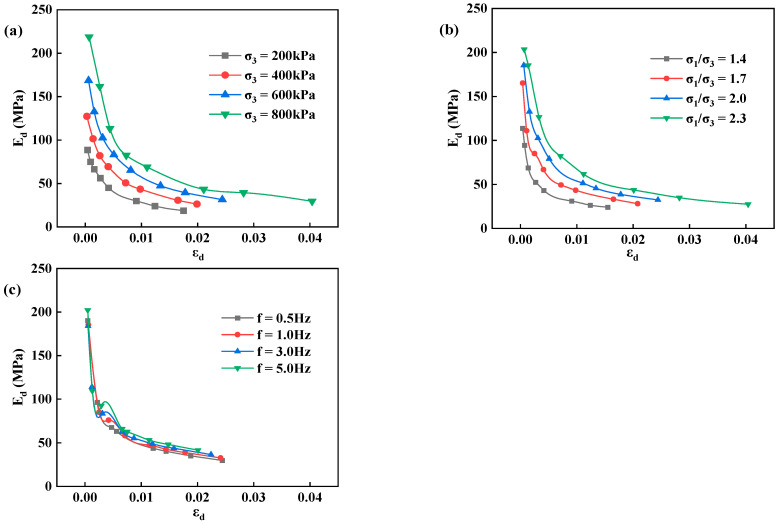
The relationship between the dynamic elasticity modulus and dynamic strain of poured asphalt concrete under different influencing factors. (**a**) confining pressure; (**b**) principal stress ratio; (**c**) vibration frequency.

Based on the test results of dynamic stress-strain, the variation pattern of dynamic elasticity modulus and the impacts of confining pressure, principal stress ratio, and vibration frequency on the curve of dynamic elasticity modulus-dynamic strain were explored, as shown in Figure 4.

Under the effects of various factors (confining pressure, principal stress ratio, and vibration frequency), the curves of dynamic elasticity modulus-dynamic strain present similar downward trends. As the load increases, the dynamic strain grows, the dynamic elasticity modulus gradually decreases with a declining magnitude, and the curve of dynamic elasticity modulus-dynamic strain becomes flat. Under the same dynamic strain, the poured asphalt concrete significantly increases with confining pressure and principal stress ratio, indicating that the two indicators prominently impact the dynamic elasticity modulus of the core wall poured asphalt concrete. Figure 3c depicts that the vibration frequency has little effect on the dynamic elasticity modulus. The greater the confining pressure and principal stress ratio, the higher the dynamic elasticity modulus of the material. The reason may be that the density and the stiffness of poured asphalt concrete enlarge with the confining pressure and principal stress ratio. The effects of increasing confining pressure and principal stress ratio on the dynamic elasticity modulus gradually drop with the dynamic strain. This is because there is a difference in the initial stress applied to the specimen at the beginning of the experiment, and the initial dynamic elasticity modulus is apparently smaller at low confining pressures than at high confining pressures. When the dynamic strain elevates to a large extent, the specimen basically enters a plastic state, and the confining pressure and principal stress ratio barely affect the dynamic elasticity modulus of poured asphalt concrete.

#### 3.1.3. Damping Ratio

**Figure 5:**
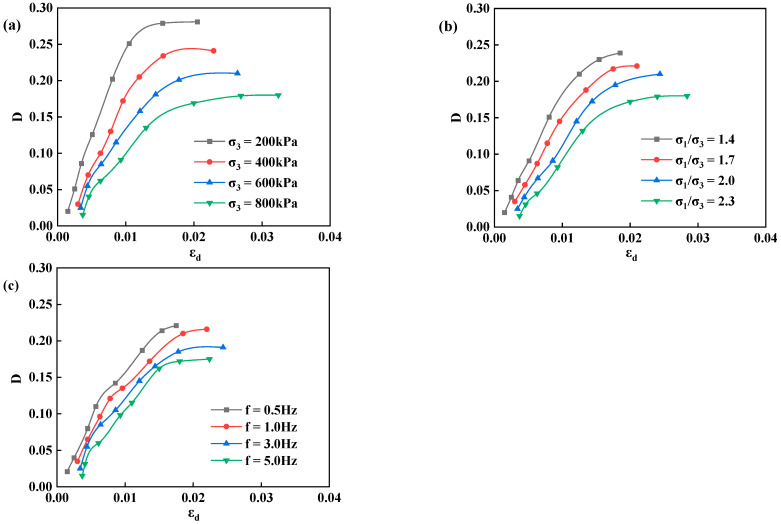
The relationship between the damping ratio and dynamic strain of poured asphalt concrete under different influencing factors. (**a**) confining pressure; (**b**) principal stress ratio; (**c**) vibration frequency.

According to the experimental plan, dynamic stress was applied in ten levels, with twenty vibrations per level. During data processing, the hysteresis loop of the 10th vibration of each level was selected for damping ratio (D) calculation. The variation pattern of damping ratio-dynamic strain of poured asphalt concrete and the effects of confining pressure, principal stress ratio, and vibration frequency on damping ratio were studied, as shown in Figure 5.

Under the influences of confining pressure, principal stress ratio, and vibration frequency, the variation patterns of the curves of damping ratio-dynamic strain of poured asphalt concrete are almost the same, and the damping ratios all increase with the dynamic strain. With a small dynamic strain at the beginning, the damping ratio of poured asphalt concrete enlarges rapidly with the dynamic strain. As the dynamic strain rises, the curve of damping ratio-dynamic strain tends to flatten. At the initial stage, the damping ratio is mildly affected by the confining pressure and principal stress ratio. As the dynamic strain of the material constantly elevates, the influences of confining pressure and principal stress ratio on the damping ratio are heightened, manifested as the damping ratio decreasing with the confining pressure and principal stress ratio. It may be attributed to the high stiffness and low damping ratio of the material under large confining pressure and principal stress ratio. The damping ratio is relatively less affected by the vibration frequency. However, as the vibration frequency increases, it presents a decreasing trend.

### 3.2. Dynamic Constitutive Model Based on the Hardin-Drnevich Model

#### 3.2.1. Research on Dynamic Elasticity Modulus

According to the dynamic characteristics test and analysis results, under the effects of confining pressure, principal stress ratio, and vibration frequency, the variation patterns of the backbone lines of dynamic stress-strain are basically identical, which is in accordance with the hyperbolic pattern. Therefore, based on the Hardin–Drnevich hyperbolic model [46,47], the dynamic constitutive relationship of poured asphalt concrete was studied.

(6) $$\sigma_{d}=\frac{\varepsilon_{d}}{c+e\varepsilon_{d}}$$

(7) $$\frac{\varepsilon_{d}}{\sigma_{d}}=c+e\varepsilon_{d}$$

(8) $$\frac{1}{E_{d}}=\frac{1}{E_{d\max}}+\frac{\varepsilon_{d}}{\sigma_{d\max}}$$

From formula (8), the relationship can be derived between dynamic elastic modulus and dynamic strain.

(9) $$E_{d}=E_{d\max}.\frac{1}{1+\frac{\varepsilon_{d}}{\varepsilon_{r}}}$$

In the formula: $\varepsilon_{r}$: the reference line strain, $\varepsilon_{r}=\sigma_{d\max}/E_{d\max}$, dimensionless; $E_{d\max}$: the maximum dynamic elasticity modulus, in $P_{a}$.

**Figure 6:**
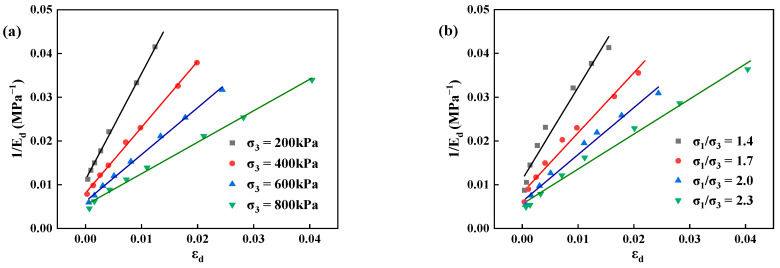

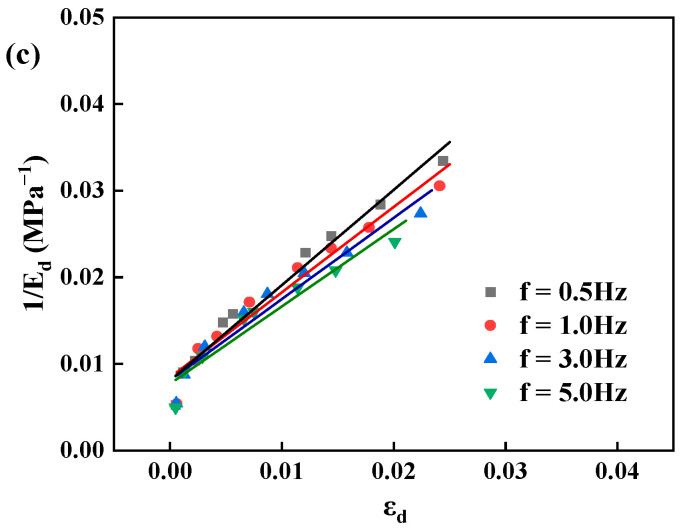
The relationship between the reciprocal of the dynamic elasticity modulus and the dynamic strain of poured asphalt concrete under different influencing factors. (**a**) confining pressure; (**b**) principal stress ratio; (**c**) vibration frequency.

From formula (9), it can be concluded that to determine the expression of dynamic elasticity modulus-dynamic strain, it is necessary to explore the maximum dynamic elasticity modulus $E_{d\max}$. Under the influences of confining pressure, principal stress ratio, and vibration frequency, the relationship between the reciprocal of dynamic elasticity modulus ($1/E_{d}$) and the dynamic strain ($\varepsilon_{d}$) can be derived from the test results according to Equation (7), as shown in Figure 6.

When the confining pressure, principal stress ratio, and vibration frequency change, the relationship between the reciprocal of the dynamic elasticity modulus and the dynamic strain of poured asphalt concrete basically follows a linear distribution. Therefore, a straight-line fitting agrees with the actual situation. The confining pressure and principal stress ratio have greater impacts on the test results than the frequency, making them the primary considerations. The intercept (c) and slope (e) can be obtained from the fitted line, from which the maximum dynamic elasticity modulus ($E_{d\max}$) and the ultimate stress ($\sigma_{d\max}$) can be derived, respectively. Under different confining pressures and principal stress ratios, the specific values of c, e, and $E_{d\max}$ are listed in Table 7.

Based on the results of on-site and indoor experiments, Hardin and Drnevich concluded a specific relationship between the maximum shear modulus and the average effective principal stress, as expressed in the following formula:

(10) $$G_{d\max}=Kp_{a}{\left(\frac{\sigma_{m}}{p_{a}}\right)}^{n}$$

In the formula: $\sigma_{m}$: the average effective stress, in $P_{a}$; K: a test experimental parameter, representing the maximum dynamic modulus coefficient, dimensionless; n: a test parameter, denoting the maximum dynamic modulus index, dimensionless; $p_{a}$: the standard atmospheric pressure, in $p_{a}$.

The correlation between the dynamic elasticity modulus $E_{d}$ and the dynamic shear modulus $G_{d}$ is shown in Equation (11).

(11) $$G_{d}=\frac{E_{d}}{2(1+\mu )}$$

According to formulas (10) and (11), the maximum dynamic elasticity modulus can be expressed as an exponential function of the average stress $\sigma_{m}$, expressed as:

(12) $$E_{d\max}=K_{1}p_{a}{\left(\frac{\sigma_{m}}{p_{a}}\right)}^{n}$$

**Figure 7:**
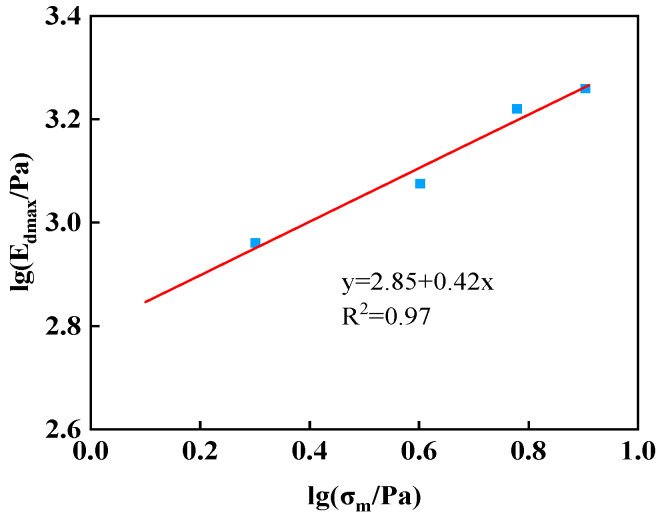
The Linear fitting results between $\mathrm{lg}(E_{d\max}/p_{a})$ and $\mathrm{lg}(\sigma_{m}/p_{a})$.

Based on the test results, the maximum dynamic elasticity modulus $E_{d\max}$ and average stress $\sigma_{m}$ were plotted in a logarithmic coordinate system, and a linear relationship of $\mathrm{lg}(E_{d\max}/p_{a})$ and $\mathrm{lg}(\sigma_{m}/p_{a})$ was fitted, as shown in Figure 7. The slope of the straight line is n, and the intercept of the straight line on the vertical axis is $\mathrm{lg}K_{1}$.

**Figure 8:**
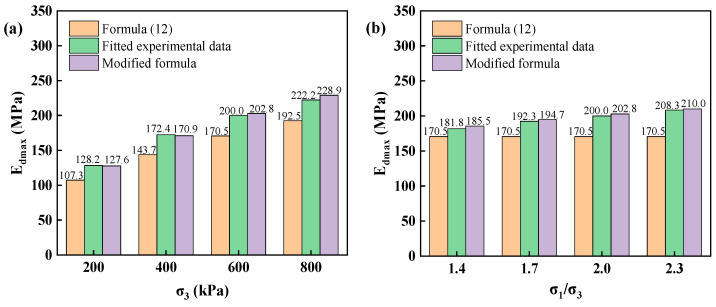
Comparison of maximum dynamic elasticity modulus of poured asphalt concrete. (**a**) confining pressure; (**b**) principal stress ratio.

According to the fitting results in Figure 8, the dynamic elasticity modulus coefficient is $K_{1}$ = 710, and the dynamic elasticity modulus index is n = 0.42. By substituting $K_{1}$ and n into formula (12), the maximum dynamic elasticity modulus can be obtained (Table 3). It evidently differs from the test fitting value in Table 3. Therefore, based on the experimental analysis results and the characteristics of the influences of confining pressure and principal stress ratio on the maximum dynamic elasticity modulus, the formula for the maximum dynamic elasticity modulus was revised, as shown in formula (13).

(13) $$E_{d\max}=K_{1}p_{a}{\left(\frac{\sigma_{m}}{p_{a}}\right)}^{n}{\left(K_{c}\right)}^{m}$$

**Figure 9:**
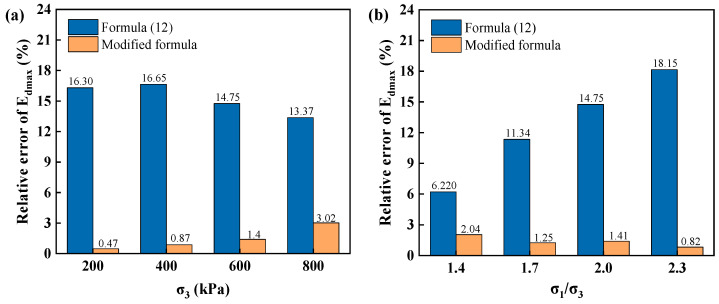
Relative error of maximum dynamic elasticity modulus of poured asphalt concrete. (**a**) confining pressure; (**b**) principal stress ratio.

Under different confining pressures and principal stress ratios, the maximum dynamic elasticity modulus of poured asphalt concrete are calculated using the modified formulas (13) and (12) and compared with the experimental fitting results. The specific data and comparison results are shown in Figure 8 and Figure 9.

Under different confining pressures and principal stress ratios, the maximum dynamic elasticity modulus of poured asphalt concrete calculated using formula (12) has large discrepancies with the experimental fitting values. The maximum relative error achieves 16.65% under different confining pressures and 18.15% with various principal stress ratios. The maximum dynamic elasticity modulus calculated using the modified formula (13) are in good agreement with the experimental fitting values, and the relative errors are significantly diminished, with maximum values of 3.02% and 2.04%, respectively.

According to formula (9) and the modified formula (13), the modified formula for the dynamic elasticity modulus of poured asphalt concrete can be expressed as:

(14) $$E_{d}=K_{1}p_{a}{\left(\frac{\sigma_{m}}{p_{a}}\right)}^{n}{\left(K_{c}\right)}^{m}.\frac{1}{1+\frac{\varepsilon_{d}}{\varepsilon_{r}}}$$

#### 3.2.2. Research on the Damping Ratio

According to the findings of Hardin and Drnevich, it can be concluded from formulas (4) and (5) that:

(15) $$\frac{D_{eq}}{D_{\max}}=1-\frac{G_{d}}{G_{d\max}}=1-\frac{E_{d}}{E_{d\max}}$$

(16) $$D_{eq}=D_{\max}\left(1-\frac{E_{d}}{E_{d\max}}\right)=D_{\max}\frac{\varepsilon_{d}/\varepsilon_{r}}{1+\varepsilon_{d}/\varepsilon_{r}}$$

Formula (16) reflects that $D_{\max}$ is the most important parameter. Therefore, it is the focus of the following discussion.

**Figure 10:**
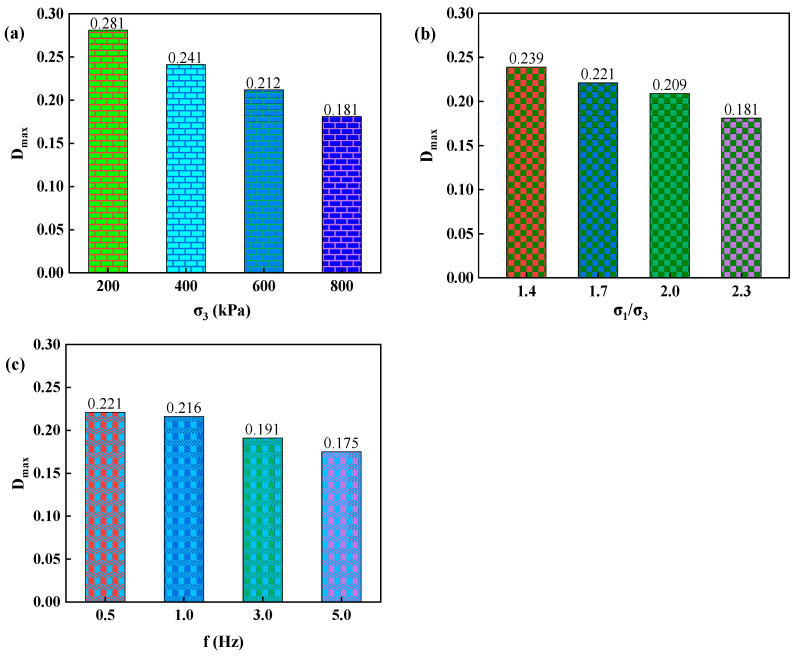
The maximum damping ratio of poured asphalt concrete under different influencing factors. (**a**) confining pressure; (**b**) principal stress ratio; (**c**) vibration frequency.

At the beginning stage of vibration, the dynamic stress and strain of poured asphalt concrete are weak. Correspondingly, the hysteresis loop area is small, representing a low damping ratio. At this time, the calculated damping ratio is inaccurate. As the dynamic stress and strain elevate, the hysteresis loop is large, and the damping ratio can be precisely determined. Therefore, when the change in the test point is small, and the damping ratio curve becomes flat, the asymptotic constant can serve as the maximum damping ratio. Based on the dynamic constitutive test results, the measured data are calculated and analyzed to obtain the maximum damping ratios under various confining pressures, principal stress ratios, and frequencies, as shown in Figure 10.

## 4. Conclusions

This study identified changing patterns of the dynamic stress-strain backbone line, dynamic elasticity modulus, and damping ratio of the poured asphalt concrete and explore the effects of various confining pressures, principal stress ratios, and vibration frequencies on the dynamic characteristics of the material. Based on the research findings, a dynamic constitutive model of poured asphalt concrete was established using the Hardin–Drnevich model.

(1) The variation patterns of the dynamic stress-strain backbone lines of poured asphalt concrete are basically the same, consistent with a hyperbolic curve. The confining pressure and principal stress ratio remarkably impact the backbone line of dynamic stress-strain of poured asphalt concrete. In contrast, the influence of vibration frequency is minor.
(2) The curves of dynamic elasticity modulus-dynamic strain of poured asphalt concrete have similar variation trends. According to the analysis of the straight line fitting results of the inverse dynamic elasticity modulus-dynamic strain, the intercept (c) and slope (e) can be obtained, from which the maximum dynamic elasticity modulus and the ultimate stress can be derived, respectively.
(3) The confining pressure and principal stress ratio have significant impacts on the maximum dynamic elasticity modulus of poured asphalt concrete. Conversely, frequency barely affects it. Subsequently, there is an evident difference between the calculated values of the original formula and the fitted values of the experimental data for the maximum dynamic elasticity modulus, with the maximum relative errors of 16.65% and 18.15%, respectively. Therefore, the expression of the maximum dynamic elasticity modulus was modified. The modified formula was used to calculate. The maximum relative errors are 3.02% and 2.04%, respectively.
(4) The variation patterns of the damping ratio-dynamic strain curves of poured asphalt concrete are basically the same under the influences of confining pressure, principal stress ratio, and vibration frequency. At the initial stage of vibration, the hysteresis loop area is small. The calculated damping ratio is imprecise. After reaching a certain extent, the curve of damping ratio-dynamic strain tends to flatten. Therefore, the asymptotic constant can serve as the maximum damping ratio.

## Figures and Tables

**Table 1:** Mix proportions of poured asphalt concrete materials.

Project	Proportion of Material Dosage (%)				
Types of materials	(9.5~19) mm	(4.75~9.5) mm	(2.36~4.75) mm	(0.075~2.36) mm	Filler dosage
Mixing ratio of mass	23	15	15	35	12

**Table 2:** Proportions of the asphalt.

Test Properties	Unit	Standard	Test Result
Penetration (25 °C,100 g, 5 s)	0.1 mm	60~80	73.9
Ductility (5 cm/min, 15 °C)	cm	≥150	159
Density (25 °C)	g/cm^3^	-	0.981
Softening point	°C	≥45	47.7
loss by mass	%	±0.8	0.031
Residual ductility (5 cm/min, 15 °C)	cm	≥80	90
Residual penetration	%	≥61	79

**Table 3:** Proportions of the coarse aggregate.

Test Properties	Unit	Standard	Test Result (2.36~4.75) mm	Test Result (4.75~9.5) mm	Test Result (9.5~19) mm
Apparent density	g/cm^3^	≥2.6	2.7	2.67	2.73
Crushing value	%	≤30	9	—	—
Water absorption	%	≤2.0	0.4	0.1	0.1
Mud content	%	≤0.5	0.4	0.3	0.1

**Table 4:** Proportions of the fine aggregate.

Test Properties	Unit	Standard	Test Result
Apparent density	g/cm^3^	≥2.55	2.68
Water absorption	%	≤2.0	1.2
Water stability level	grade	≥6	9
Mud content	%	≤2	0.6

**Table 5:** Proportions of the filler (cement).

Test Properties	Particle Size Range	Standard	Test Result
Fineness (%)	<0.6 mm	100	100
	<0.15 mm	>90	99.72
	<0.075 mm	>85	96.98
Apparent density (g/cm^3^)		≥2.5	3.07
Hydrophilicity coefficient		≤1.0	0.79
Rate of water conten		≤0.5	0.02

**Table 6:** Testing plan of dynamic characteristics.

Test Group Number	Confining Pressures (kPa)	Principal Stress Ratio	Vibration Frequencies (Hz)
WY1	200	2.0	1.0
WY2	400	2.0	1.0
WY3	600	2.0	1.0
WY4	800	2.0	1.0
YLB1	600	1.4	1.0
YLB2	600	1.7	1.0
YLB3	600	2.0	1.0
YLB4	600	2.3	1.0
PL1	600	2.0	0.5
PL2	600	2.0	1.0
PL3	600	2.0	3.0
PL4	600	2.0	5.0

**Table 7:** Test parameters and the maximum dynamic modulus of elasticity under different confining pressures and principal stress ratios.

Test Group Number	Confining Pressures (kPa)	Principal Stress Ratio	Vibration Frequencies (Hz)	Parameters		Fitted Value by Experimental Data $E_{d\max}$ (MPa)
				c	e	
WY-1	200	2.0	1.0	0.0078	2.4915	128.2
WY-2	400	2.0	1.0	0.0058	1.4443	172.4
WY-3	600	2.0	1.0	0.005	1.0969	200.0
WY-4	800	2.0	1.0	0.0045	0.8366	222.2
YLB-5	600	1.4	1.0	0.0055	1.6541	181.8
YLB-6	600	1.7	1.0	0.0052	1.3762	192.3
YLB-7	600	2.0	1.0	0.0050	1.1524	200.0
YLB-8	600	2.3	1.0	0.0048	0.9815	208.3

## Data Availability

The raw data supporting the conclusions of this article will be made available by the corresponding author on request.
